# A role for BRG1 in the regulation of genes required for development of the lymphatic system

**DOI:** 10.18632/oncotarget.18976

**Published:** 2017-07-04

**Authors:** Ajeet Pratap Singh, Julie Foley, Arpit Tandon, Dhiral Phadke, H. Karimi Kinyamu, Trevor K. Archer

**Affiliations:** ^1^ Chromatin and Gene Expression Section, Epigenetics and Stem Cell Biology Laboratory, National Institute of Environmental Health Sciences, National Institutes of Health, Research Triangle Park, North Carolina, USA; ^2^ Special Techniques Group, Cellular and Molecular Pathology Branch, National Institute of Environmental Health Sciences, National Institutes of Health, Research Triangle Park, North Carolina, USA; ^3^Sciome.com, National Institute of Environmental Health Sciences, National Institutes of Health, Research Triangle Park, North Carolina, USA; ^4^ Present address: Cornell University, College of Veterinary Medicine, Ithaca, New York, USA

**Keywords:** BRG1, LYVE1, development, lymphatic, lymphedema

## Abstract

Lymphatic vasculature is an important part of the cardiovascular system with multiple functions, including regulation of the return of interstitial fluid (lymph) to the bloodstream, immune responses, and fat absorption. Consequently, lymphatic vasculature defects are involved in many pathological processes, including tumor metastasis and lymphedema. BRG1 is an important player in the developmental window when the lymphatic system is initiated. In the current study, we used tamoxifen inducible *Rosa26CreERT2-BRG1^floxed/floxed^* mice that allowed temporal analysis of the impact of BRG1 inactivation in the embryo. The *BRG1^floxed/floxed/Cre-TM^* embryos exhibited edema and hemorrhage at embryonic day-13 and began to die. BRG1 deficient embryos had abnormal lymphatic sac linings with fewer LYVE1 positive lymphatic endothelial cells. Indeed, loss of BRG1 attenuated expression of a subset of lymphatic genes *in-vivo*. Furthermore, BRG1 binds at the promoters of *COUP-TFII* and *LYVE1*, suggesting that BRG1 modulates expression of these genes in the developing embryos. Conversely, re-expression of BRG1 in cells lacking endogenous BRG1 resulted in induction of lymphatic gene expression *in-vitro, suggesting* that BRG1 was both required and sufficient for lymphatic gene expression. These studies provide important insights into intrinsic regulation of BRG1-mediated lymphatic-gene expression, and further an understanding of lymphatic gene dysregulation in lymphedema and other disease conditions.

## INTRODUCTION

The lymphatic system plays key role in development and in regulating tissue fluid, thereby maintaining tissue homeostasis [1, 2]. Its dysfunction results in life threatening diseases such as lymphedema, lymphangiectasia, lymphangioma, and lymphatic dysplasia [3]. Furthermore, tumor lymphangiogenesis has been implicated in cancer metastasis [4]. Blood vessels originate during embryonic development from mesodermally derived endothelial cell precursors. Endothelial sprouting and splitting contributes to the formation of mature network of blood vessels. Blood and lymphatic vessels develop from the same cell lineage but they perform very distinct and essential circulatory functions [1]. Subsequent to blood vessel formation, the lymphatic vasculature appears, perhaps indicating that lymphatics might have a blood vasculature origin. However, the molecular factor(s) that regulate this initial stage of lymphatic competence and establishment of the lymphatic system, remain elusive. A clear nexus of signal transduction pathways, such as the VEGF-C/VEGFR-3 system and transcription factors including, *PROX-1, SOX18* and *COUP-TFII* were shown to be critical for this process [1, 5].

BRG1 is the central catalytic subunit of the SWI/SNF chromatin-remodeling complex [6–9]. Numerous studies have placed SWI\SNF complex and its subunits at the nexus of transcriptional networks critical for many biological and disease processes [10–13]. BRG1 is required for peri-implantation development and heart formation [14–19]. More recently we established that BRG1 is highly and ubiquitously detectable in peri-gastrulation stage embryos. Global inactivation of BRG1 during peri-gastrulation causes growth retardation and ultimately embryonic mortality [20].

To explore the distinctive functional role(s) of BRG1, we inactivated BRG1 in the whole embryo during mid-to-late gestation stages of development. Embryos lacking BRG1 function (s) show a range of aberrant phenotypes that are typically associated with lymphatic defects and diminished expression of a sub-set of lymphatic genes, suggesting a novel and an essential role for BRG1 in the lymphatic system during development.

## RESULTS AND DISCUSSION

### BRG1 is detected across all stages of development

**Figure 1:**
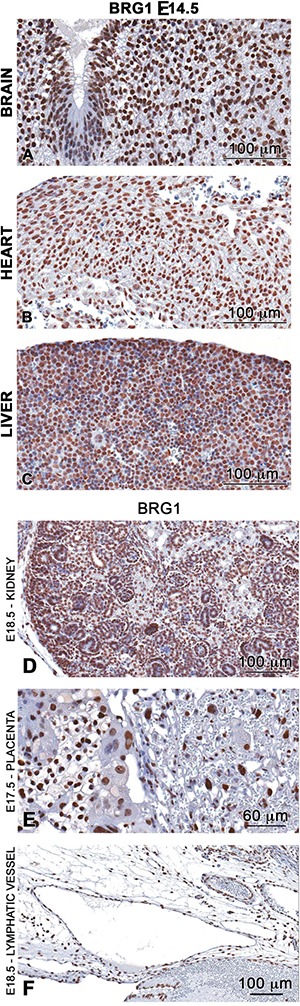
BRG1 is ubiquitously expressed in the developing embryo Immunohistochemistry analysis demonstrates high abundance nuclear staining of BRG1 (brown stain) in brain (**A**), heart (**B**) liver (**C**) kidney (**D**) placenta (**E**) and in endothelial cells of the lymphatic vessel (**F**) of developing wild-type embryos.

Previous reports established that BRG1 is essential for early embryogenesis and heart formation [16, 18, 21, 22]. Unlike transcription factors, which can display differential spatial-temporal expression patterns and thereby provide cellular specificity, BRG1 is ubiquitously expressed in peri-gastrulation stage embryos and acts as a global regulator of transcription [20]. To explore the role of BRG1 in later stages of development, we began by examining the spatiotemporal localization of BRG1 protein in mid-to-late gestation stage embryos. Immunohistochemistry analysis showed positive BRG1 staining localized to the cell nucleus of all organs examined including brain, heart, liver, kidney, placenta and lymphatic vessels (lymphatic endothelial cells) (Figure 1A–1F). The distribution of BRG1 staining was homogenous in the nuclei across a range of cell types in brain, heart, liver, kidney, placenta, and vasculature endothelial cells. This pattern was repeated over time across different developmental stages (E11.5 to E18.5) (Supplementary Figure 1), suggesting that BRG1 is expressed throughout development. Ubiquitous and abundant expression of BRG1 raised the possibility that BRG1 function (s) in a wide spectrum of developmental processes for embryonic growth and maintenance of tissue homeostasis beyond the early development stages when embryonic cells undergo rapid proliferation and differentiation.

### BRG1 is essential during the mid-to-late stages of embryonic development

To study the implications of the loss of BRG1 function, we used a tamoxifen inducible *Cre-lox* system to identify roles of BRG1 in mid-to-late gestation stages of fetal development. The *BRG1^floxed/floxed^* dams were time mated with *BRG1^floxed/floxed/R26CreER-TM^* sires, pregnant dams were intraperitoneally injected with tamoxifen at E10.5 days post coitum (dpc.), and embryos were collected at E13.5 dpc. Alternatively, time mated pregnant mice received two consecutive doses at E12.5 and 13.5, and embryos were collected at E16.5dpc for morphological analysis. The *BRG1^floxed/floxed/R26CreER-TM^* (hereafter referred as *BRG1^d/d^*; embryos deficient for BRG1) embryos exhibited multiple morphologically distinct phenotypes when compared with *BRG1^floxed/floxed^* (hereafter referred as *BRG1^fl/fl^*; wild-type) embryos. (Supplementary Figure 2)

**Figure 2:**
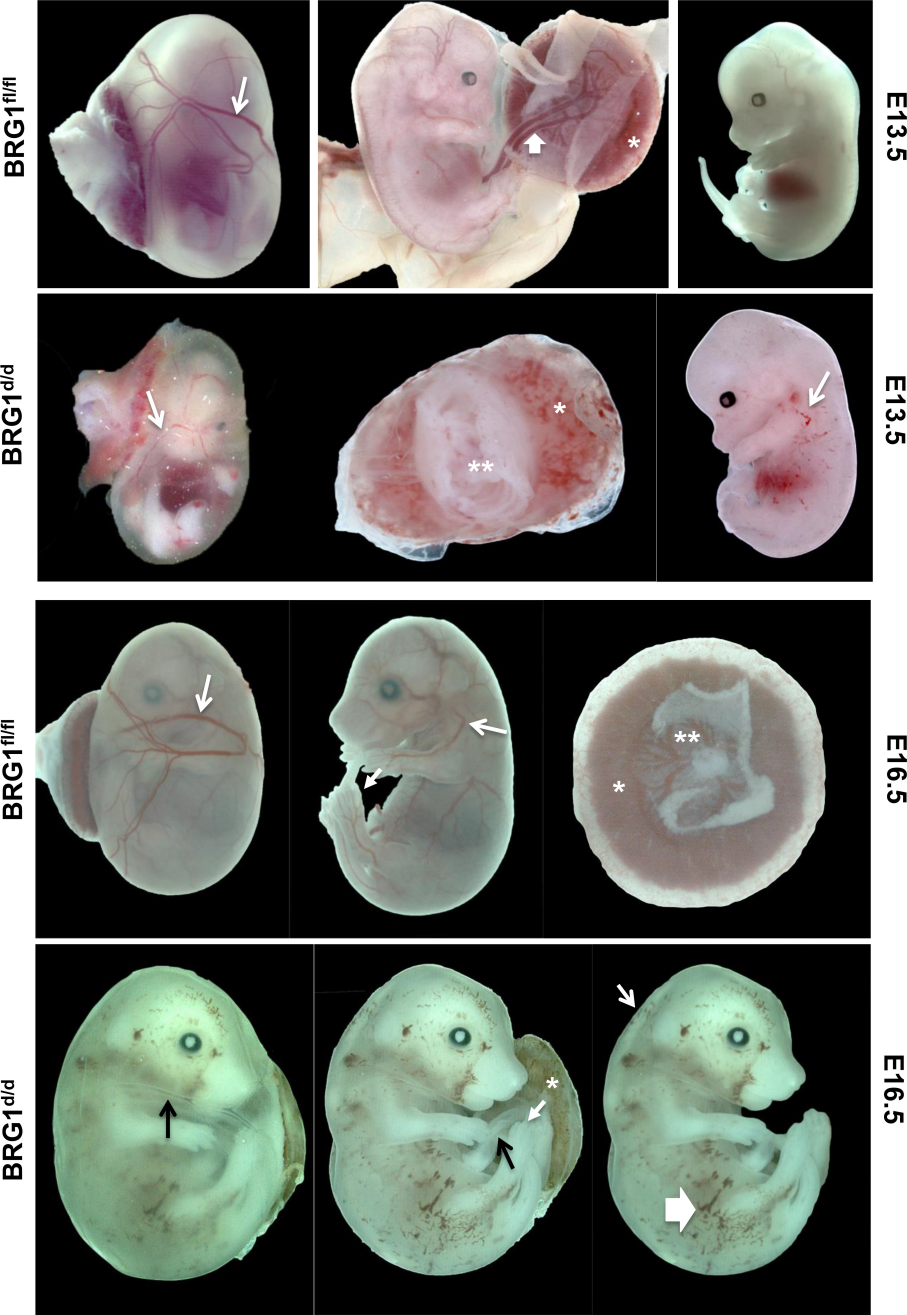
BRG1 is necessary during mid-to-late stage embryogenesis Temporal deletion of *BRG1^fl/fl^* alleles results multiple defects and led to embryonic mortality. The *BRG1^fl/fl^* pregnant female mice time mated with male *BRG1^fl/fl^/R26CreER-TM*mice were dosed with tamoxifen (100 mg/kg body weights) either at E10.5 (single dose) or at E12.5 and 13.5 (two consecutive doses). Embryo phenotype was then evaluated at E13.5 and 16.5 respectively. In E13.5 *BRG1^fl/fl^* embryos, arrows indicate normal blood vessels. In the E13.5 *BRG1^d/d^* embryos, arrows indicate defective blood vessels and hemorrhages with the asterisk indicating anemic placenta. In E16.5 *BRG1^d/d^* embryos, thin arrow indicates edema and thick arrow indicates hemorrhages.

In embryonic day (E) 13.5 *BRG1^fl/fl^* embryos, the appearance of the conceptus was within normal limits for all litters collected (Figure 2). Normal patterning of the vitelline vessels (arrow) was observed on the exterior surface of the yolk sac. All vessels were filled with red blood cells. Removal of the extra embryonic membranes showed no abnormalities with the umbilical vessels (wide arrow). The placenta (asterisk) was normal in shape, color, and size. The developing embryo possessed features of gestation day 13.5 as characterized by clear indentation of the anterior footplate, while the posterior footplate was still webbed and absent of indentations. In contrast, conditional inactivation of BRG1 on E10.5 resulted in many irregularities on E13.5 (Figure 2). The vitelline vessels were present, but it was difficult to grossly distinguish if the vessel remodeling was affected (i.e. angiogenesis of the vitelline artery and veins). The vessels were small without signs of homogenous filling with red blood cells (RBCs, arrow). In addition, RBCs were not always visible in the umbilical vessels (double asterisk). The placenta was small, pale and the fetal surface was irregular and mottled. Embryos collected at this time point were smaller and paler than the WT and had blood filled spaces (arrow) on the surface of the embryo. Typically, these blood-filled spaces were localized to the lateral aspects of the embryo. The majority of the embryos were edematous with the degree ranging from mild to severe.

At E16.5 *BRG1^fl/fl^*, the appearance of the conceptus was within normal limits for all litters collected (Figure 2). Normal patterning of the vitelline vessels (arrow) was observed on the exterior surface of the yolk sac (arrow). RBCs filled the vitelline vessels. Removal of the extra embryonic membranes showed no abnormalities with the umbilical vessels. The placenta (asterisk) was normal in shape and size. The labyrinth (lab) and junctional zone (jz) were morphologically normal (*, asterisk). Features of gestation day 16.5 embryos were characterized by the end phalanges of forelimb and hind limb. The forelimb phalanges were nearly parallel and joined by skin proximally, but not near the distal tips. The hind limb phalanges were more divergent. In contrast, *BRG1^d/d^* conceptus at E16.5 revealed many obvious abnormalities (Figure 2; →, arrows). The vitelline vessels were present, but it was difficult to grossly distinguish if vitelline vessel remodeling was affected. The vessels were thin without homogenous filling with RBCs (arrow). In addition, RBCs were not always visible in the umbilical vessels (arrow). The placenta was small, pale and the fetal surface was irregular and mottled (asterisk). Embryos were pale and exhibited blood filled spaces (wide arrow) on the surface of the embryo. In the E16.5 embryos the blood-filled spaces appeared to be more extensive than in the E13.5. The majority of the embryos were edematous with the degree ranging from moderate to severe.

### BRG1-deficient embryos showed abnormalities associated with lymphatic dysfunction

As development proceeds the lymphatic and blood vasculatures spread out in the whole body, and remain separated from each other. The adapter protein SLP76 and the tyrosine kinase SYK are mainly expressed by hematopoietic cells and have been shown to play a role in separating of blood and lymphatic vasculatures [23]. Indeed, mice lacking either SLP76 or SYK exhibit abnormal blood-lymphatic connections; embryonic hemorrhage and arteriovenous shunting [23].

**Figure 3:**
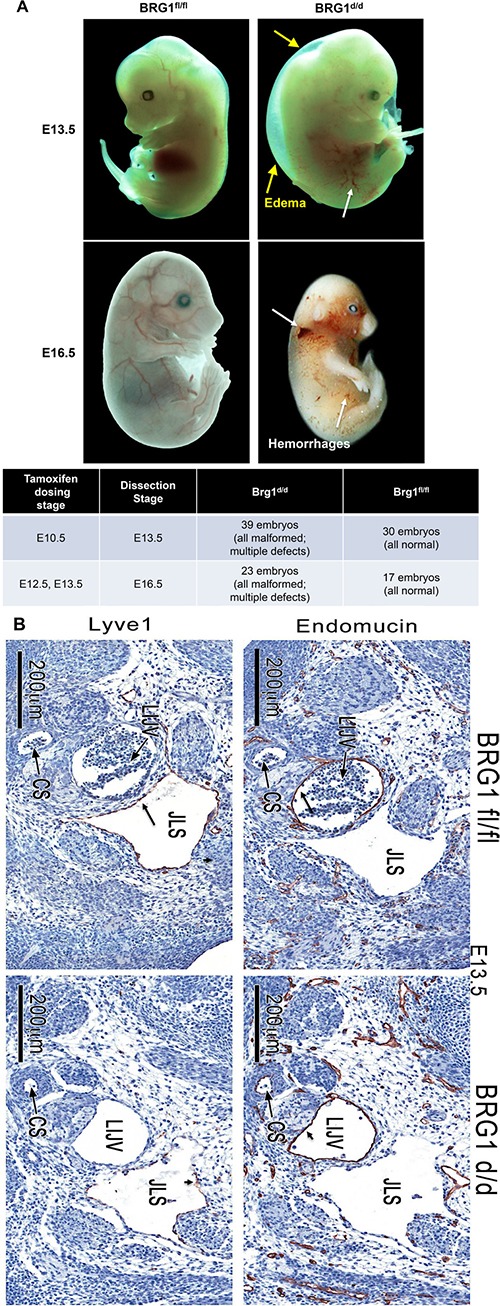
*BRG1-*deficient embryos show hemorrhages (**A**) Panels show gross morphology of the *BRG1^fl/fl^* and *BRG1^d/d^* embryos at the indicated developmental stages. White arrows – edema; yellow arrows – hemorrhage, lower panel of table shows number of *BRG1^d/d^* and *BRG1^fl/fl^* embryos that were analyzed for gross phenotype (**B**) Immunohistochemistry staining for ENDOMUCIN and LYVE1 identifies the jugular vein (jv) and the lymph sacs (ls) specifically in E13.5 *BRG1^fl/fl^* and *BRG1^d/d^* embryos. CS; carotid sinus, JLS; jugular lymphatic sac, LIJV; left internal jugular vein.

Inactivation of BRG1 during mid-to-late stage embryonic development resulted in a range of abnormal phenotypes associated with defective lymphatic and blood vasculatures [24–27]. Most *BRG1^d/d^* embryos showed gross subcutaneous edema and swelling at E13.5 and 16.5 (Figure 3A, Supplementary Figure 2), and subsequently fetal mortality in all *BRG1^d/d^* embryos, as described previously by other researchers in different mouse models [24, 28, 29]. An array of abnormal phenotypes such as formation of subcutaneous edema, cerebral hemorrhage near the cardinal vein, and hemorrhaging throughout the body (Figure 3A, Supplementary Figure 2) were mostly seen in BRG1 mutant embryos. Blood vessels and the lymph sac were formed in *BRG1^fl/fl^* embryos; but smaller size of blood vessels and abnormally patterned lymphatic sacs were observed in *BRG1^d/d^* embryos (Figure 3B). ENDOMUCIN, also known as endothelial SIALOMUCIN, which interferes with the assembly of focal adhesion complexes and inhibits interaction between cells and the extracellular matrix, staining was comparable in the left internal jugular vein of both *BRG1^fl/fl^* and *BRG1^d/d^* embryos (Figure 3B, right panel upper and lower). Strikingly, staining for LYVE1, a cell surface protein expressed specifically by lymphatic endothelial cells [30, 31], co-localized with the lining of the lymphatic endothelial cells in lymphatic sac of the *BRG1^fl/fl^* embryos (Figure 3B; upper left panel). In contrast, diminished LYVE1 staining or loss of lymphatic endothelial cells were observed in the lymphatic sac of *BRG1^d/d^* embryos (Figure 3B; lower left panel). This finding suggests a role for BRG1 in developmental of lymphatic system.

Earlier studies have established the homeobox transcription factor PROX1 as a critical regulator of a number of lymphatic genes, including cell fate specification and maintenance of the LYVE1 positive lymphatic endothelial cells (LECs), and development of the murine lymphatic vasculature [32, 33]. To ascertain if BRG1 is necessary for PROX1 expression in lymphatic precursors *in-vivo*, we examined the localization pattern of PROX1 in *BRG1^d/d^* embryos, in comparison to *BRG1^fl/fl^* embryos. By E13.5, linear groups of PROX1-positive cells were clearly visibly? visible lining the primary lymph sac in *BRG1^fl/fl^* embryos (Supplementary Figure 3, arrows). In contrast, lymphatic endothelial cells were mostly absent in *BRG1^d/d^* embryos (Supplementary Figure 3), although the staining intensity for PROX1 was comparable in the remaining cells to that seen in the lymphatic sac of the *BRG1^fl/fl^* embryo (Supplementary Figure 3, arrows). *BRG1^fl/fl^* embryos contained a round, spherical jugular vein completely filled with red blood cells. However, an abnormal structure lacking red blood cells was present in *BRG1^d/d^* embryos. These observations establish that the lack of BRG1 function results in a dysregulated lymphatic system from the pool of vascular endothelial cell precursors during mouse embryogenesis, confirming the pivotal role of BRG1 in the development of this system.

### Specific sets of lymphatic genes were repressed in *BRG1^d/d^* embryos

**Figure 4:**
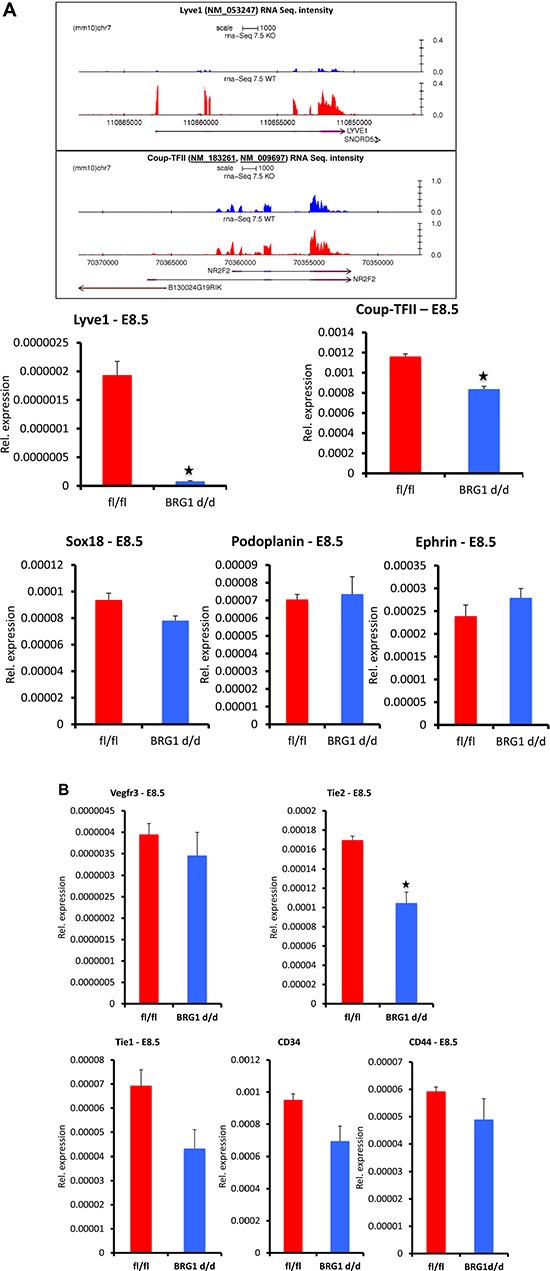
Expression level (mRNA) of a subset of lymphatic markers is attenuated in COUP-TFII (*NR2F2*) *BRG1^d/d^* embryos (**A**) Upper panel, RNA sequencing analysis shows gene track and RNA expression signal of the *LYVE1* and *COUP-TFII* in *BRG1^fl/fl^* vs. *BRG1^d/d^* embryos at E7.5. Lower panel, *LYVE1* and *COUP-TFII* expression level is significantly reduced in *BRG1^d/d^* E8.5 embryos, as assessed by quantitative RT–PCR. *PODOPLANIN*, *SOX18* and *EPHRIN* expression in *BRG1^d/d^* embryos is comparable to *BRG1^fl/fl^* embryos at E8.5. (**B**) Expression level of blood endothelial markers; *TIE1*, *VEGFR3*, *CD34* and *CD44* was reduced non-significantly in *BRG1^d/d^* embryos in comparison to *BRG1^fl/fl^* embryos. *TIE2* expression was reduced significantly in *BRG1^d/d^* embryos. The experiment was performed with at least three biological replicates *P*-value < 0.05.

Endothelial cells acquire LEC-specific gene signature during early development, leading to the subsequent assembly of the lymphatic vascular network. Thus, expression of LEC related genes are critical for both lymphatic network formation and function during mid-to-late stage of development, following their role in LEC fate specification [3]. To gain molecular insights into BRG1 dependent LEC related gene expression in *BRG1^d/d^* embryos, we searched for genes related to the lymphatic system in our genome wide RNA sequencing and microarrays analyses data sets of E7.5 and E8.5 *BRG1^d/d^* vs. *BRG1^fl/fl^* embryos [20]. The LEC related genes were differentially expressed in E7.5 and E8.5 *BRG1^d/d^* embryos prior to the appearance of the abnormal lymphatic phenotype and this could explain the defective defective function of lymphatic system during mid-to-late stages of *BRG1^d/d^* embryos. We found several lymphatic and blood endothelial-specific genes that were differentially expressed in *BRG1^d/d^* embryos compared to *BRG1^fl/fl^* embryos. Of particular interest, we found that *LYVE1* and *COUP-TFII (NR2F2)*, which are known lymphatic endothelial-specific marker genes and initially expressed during lymphatic endothelial cell specification in developing embryo [8, 34], were significantly down regulated (*LYVE1˜* 9.7 fold, *COUP-TFII* ˜2.6 fold) in *BRG1^d/d^* embryos (Table 1; Figure 4A; upper panel, shows gene track of RNA sequencing signals). Additionally, we found several other genes associated with lymphatic system and immune responses were differentially expressed in microarray analysis of E8.5 *BRG1^d/d^* embryos (Table 1). As expected, *PROX1*, a transcription factor, that is involved in cell fate determination and function as a regulatory protein in the lymphatic system, was not detected in E7.5 and E8.5 global gene expression analyses because it is known not to be expressed at these early stages [35]. Using quantitative RT-PCR, we validated changes in expression of lymphatic genes by evaluating the mRNA level at E8.5 in *BRG1^d/d^* vs. *BRG1^fl/fl^* embryos. QPCR analysis shows attenuated expression level of selected lymphatic endothelial-related genes including *LYVE1* and *COUP-TFII* in *BRG1^d/d^* embryos vs. *BRG1^fl/fl^* embryos (Figure 4A; lower panel), consistent with the genome wide transcriptome analyses. In addition, we also observed that immune response genes such as SWI/SNF2-related *HELLS* (SMARCA6), *LY6E*, and *LY6I* were also altered in the *BRG1^d/d^* embryos (Table 1, Supplementary Figure 4). These immune responsive genes were previously reported to regulate the expansion or survival of lymphoid cells [36]. Surprisingly, deletion of *BRG1* did not change expression of other known lymphatic genes including *SOX18*, *PODOPLANIN* and *EPHRIN* (Figure 4A; lower panel) and general vascular endothelial cell differentiation markers such as *VEGFR3*, *CD34* and *CD44* (Figure 4B). This finding suggests that BRG1 expression is crucial for LEC maintenance, patterning and function of lymphatic vessels in mid-to-late embryos. However, vascular endothelial related genes such as *TIE2* were significantly decreased in E8.5 *BRG1^d/d^* embryos accompanied by a lessor reduction of *TIE1* (Figure 4B). *VCAM* expression was also significantly decreased in *BRG1^d/d^* vs. *BRG1^fl/fl^* embryos (Supplementary Figure 4, lower panel). Therefore, BRG1 stimulates expression of a subset of known lymphatic marker genes during the differentiation of cells along a vascular endothelial-specific pathway during mouse development.

**Table 1:** Lymphatic endothelial related genes differentially expressed in Brg1d/d vs. Brg1fl/fl embryos

Accession #	Primary Sequence Name	7.5_Brg1 cKO/7.5_Control Fold Change	7.5_Brg1 cKO/7.5_Control ANOVA *p*-value
**NM_053247**	Lyve1	−9.74723123	5.97E-09
NM_009868	Cdh5	−2.982166891	3.71E-07
**Accession #**	**Primary Sequence Name**	**8.5_Brg1 cKO/8.5_Control Fold Change**	**8.5_Brg1 cKO/8.5_Control ANOVA** ***p*-value**
NM_019391	Lsp1	−2.9313	0.0487
**NM_009697**	**Nr2f2**	**−2.5809**	**0.0487**
**Z11886**	**Notch1**	**−2.5614**	**0.0476**
NM_007937	Epha5	−2.4067	0.0155
NM_008511	Lrmp	−2.3861	0.033
NM_008529	Ly6e	−2.3109	0.0079
AK021284	Mll5	−2.0003	0.0329
**NM_008234**	**Hells**	**−1.6854**	**0.0289**
NM_133743	Lypd3	3.9961	0.0049
NM_033601	Bcl3	3.0254	0.0398
NM_020498	Ly6i	2.8415	0.0286
ENSMUST00000029673	Efna3	2.6045	0.0274
AK029978	Prox2	2.5298	0.0362
NM_001033281	Prdm6	1.8703	0.0216
NM_007548	Prdm1	1.6187	0.0249

Green: Repressed genes; Red: Induced genes.

### Key lymphatic genes *LYVE1* and *COUP-TFII* are direct targets of BRG1

**Figure 5:**
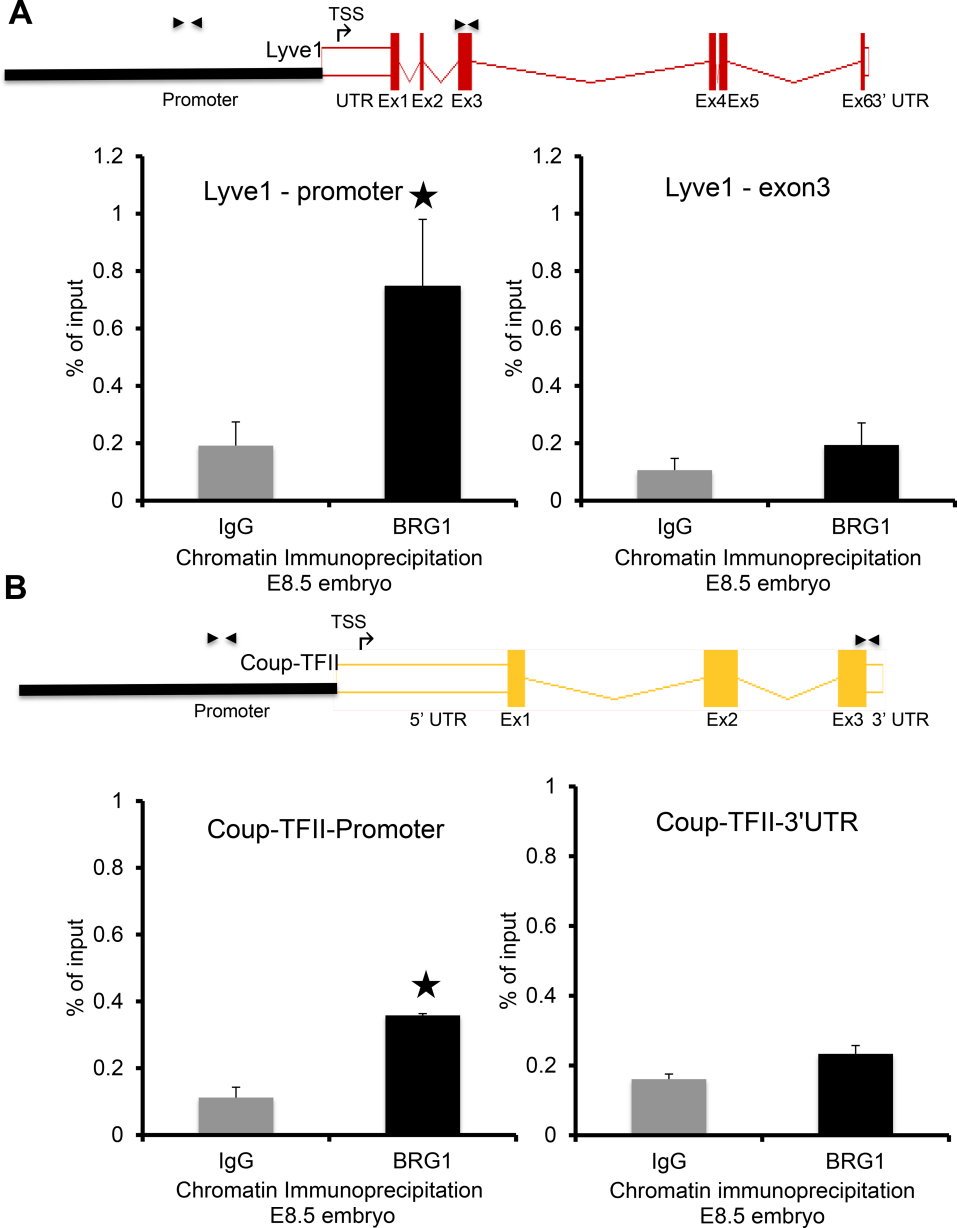
BRG1 is bound at the promoters of key lymphatic genes: Quantitative PCR was performed with primers designed from promoter and coding regions of the target genes (**A**) *LYVE1* and (**B**) *COUP-TFII* after chromatin immunoprecipitation (ChIP) with BRG1 antibody and IgG using E8.5 embryos. The schematics above the QPCR data show ChIP primer locations upstream of transcription start site (TSS) and within an exon of the *LYVE1* and *COUP-TFII* genes. The experiment was performed with at least three biological replicates *P*-value < 0.05.

To confirm the regulatory function of BRG1 on modulating expression of *LYVE1* and *COUP-TFII*, chromatin immunoprecipitation experiments were performed using chromatin from E8.5 wild type mouse embryos, a developmental stage when endogenous expression of *LYVE1* and *COUP-TFII* is detectable [34]. DNA fragments were immunoprecipitated using anti-BRG1 antibody and control IgG, purified and tested for the presence of the BRG1 binding on the *LYVE1* and *COUP-TFII* promoters relative to the coding region. A clear enrichment of BRG1 over IgG was detected by QPCR on the tested loci of *LYVE1* and *COUP-TFII* promoter, confirming that BRG1 protein binds on the *LYVE1* and *COUP-TFII* promoter (Figure 5A–5B, left graphs). However, BRG1 binding was substantially lower at the coding region of the *LYVE1* and *COUP-TFII* genes (Figure 5A–5B, right panels). Taken together, these observations are consistent with the notion that BRG1 mediated transcriptional regulation during development is required for lymphatic system.

### BRG1 modulates expression of subset of lymphatic genes in cancer cells

**Figure 6:**
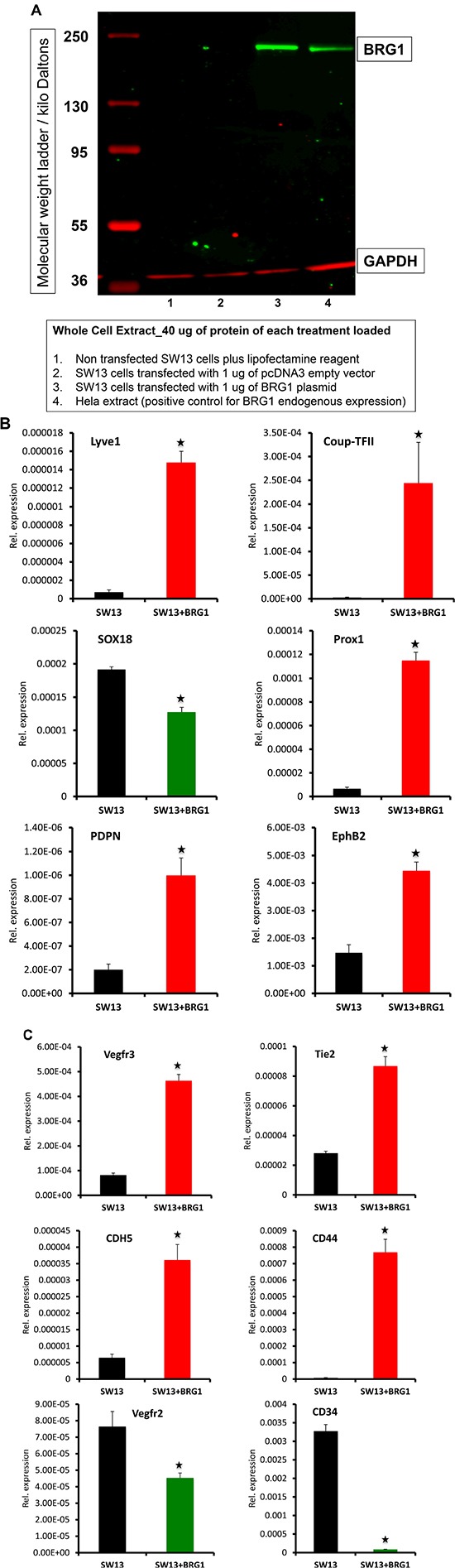
Transient expression of BRG1 in SW13 cells affects the expression of lymphatic and blood vascular endothelial related genes SW13 cells lacking endogenous expression of BRG1 were transiently transfected with a control empty vector or a plasmid containing full-length BRG1 cDNA. Twenty-four-hour post transfection, expression level of the lymphatic markers: *LYVE1*, *COUP-TFII*, *PROX1*, *SOX18*, *EPHB2*, *PDPN*, and *VEGFR3* and blood vascular endothelial markers: *TIE2*, CDH5, CD44, *VEGFR2* and CD34 were assessed by quantitative RT-PCR. The experiment was performed with at least three biological replicates. Data are means ± SEM of replicates. *P*-value < 0.05.

To investigate further the mechanism(s) of BRG1-dependent expression of lymphatic marker genes, we used cultured non-lymphatic adrenal gland/cortex derived SW13 cell line, which lack endogenous expression of BRG1 and the BRG1 dependent genes such as *CD44* [37]. SW13 cells were transiently transfected for 24hrs with a BRG1 expression plasmid, and BRG1 protein and lymphatic marker mRNA expression analyzed by western blot and quantitative RT-PCR respectively (Figure 6A–6C). Transient expression of BRG1 in SW13 cells resulted in substantial up regulation of the lymphatic genes such as *LYVE1, COUP-TFII, PROX1, EPHB2, PDPN* and *VEGFR3* (Figure 6B, 6C). Surprisingly, *SOX18* expression was repressed in SW13 cells expressing BRG1 (Figure 6B), while the expression level of the blood vascular endothelial genes *TIE2*, *CDH5* and canonical BRG1 target gene *CD44* were increased (Figure 6C). In contrast, the expression levels of *VEGFR2* and *CD34* were repressed by BRG1 overexpression (Figure 6C). Thus, BRG1 specifically enhances a subset of lymphatic endothelial genes, which further supports its potential role in promoting the lymphatic endothelial differentiation program in development and in lymphangiogenesis within tumors [38, 39].

To extend these studies we used an *in vitro* tube/vessel formation assay with a human umbilical vein endothelial cell (HUVEC) culture system to evaluate a functional role for BRG1. We observed that BRG1 silencing disrupted the ability of HUVECs to form tube/vessel structures. HUVECs transfected with siRNA targeting BRG1 showed disorganized tube formation and less tubes per seeded cell population compared to cells transfected with scrambled control siRNA (Supplementary Figure 5).

In summary, we report that BRG1 function is required for the differentiation of lymphatic endothelial progenitor cells from blood vascular precursors in the developing embryo. BRG1 is essential for growth and maintenance of LECs in developing embryo and its deficiency contributes to a wide array of lymphatic defects and mortality. BRG1 overexpression in non-lymphatic SW13 cells resulted in the induction of a spectrum of lymphatic marker genes, a result that further supports the hypothesis that BRG1 activates expression of lymphatic genes in development. Mechanistically, BRG1 both directly and indirectly regulates genes that are critical for lymphatic system in the developing embryo [10, 11, 40]. Specifically, BRG1 directly binds on the promoters of key lymphatic genes such as the transcription factor *COUP-TFII* and target gene such as *LYVE1*.

Many critical developmental events during embryogenesis and subsequent organogenesis employ combinations of transcription factors, signal transduction pathways, and chromatin remodeling proteins [1, 19, 41]. The role of BRG1 in promoting lymphatic endothelial cell specification is consistent with SWI/SNF components acting as developmental switches/fine tuners. Moreover, BRG1 is clearly determinative in a number of developmental processes–early embryogenesis, cardiovascular system, and neurogenesis–and is likely to depend on context-specific partner proteins and/or cofactors [14, 16, 19, 42]. Results of this study provide novel insights regarding development of the lymphatic system and the relatively unexplored aspects of chromatin/epigenetic regulation in this system. Further studies will need to focus on defining the cellular and molecular milieu in which BRG1 operates to activate lymphatic transcription, and may reveal new strategies for therapeutic stimulation or suppression of lymphangiogenesis [11].

## MATERIALS AND METHODS

Materials and Methods are available in the online-only Data Supplement.

## SUPPLEMENTARY MATERIALS FIGURES



## Abbreviations

BRG1: Brahma-related gene 1
COUP-TFII: Chicken ovalbumin upstream promoter transcription factor 2
DEG: Differentially expressed genes
LECs: Lymphatic endothelial cells
LYVE1: Lymphatic vessel endothelial hyaluronan receptor 1
PROX1: Prospero Homeobox 1
SMARCA4: SWI/SNF Related, Matrix Associated, Actin Dependent Regulator Of Chromatin, Subfamily A, Member 4
SOX18: Sex Determining Region Y-Box 18
SWI/SNF: SWItch/Sucrose Non-Fermentable
VEGF: Vascular endothelial growth factor
VEGFR: Vascular Endothelial Growth Factor Receptor
