# Supplementary material for: A role for BRG1 in the regulation of genes required for development of the lymphatic system
Source: Oncotarget. 2017 Jul 4;8(33):54925–38. doi: 10.18632/oncotarget.18976 (PMC5589631; doi:10.18632/oncotarget.18976)
Supplement: Supplementary file 1 [file oncotarget-08-54925-s001.pdf]

A role for BRG1 in the regulation of genes required for development of the lymphatic system

SUPPLEMENTARY MATERIALS

MATERIALS AND METHODS

Inducible Brg1 knockout mice

The generation of an inducible *Brg1*$^{floxed/floxed}$ conditional knockout mouse model has been previously described [1]. In brief mice harboring the floxed Brg1 allele were bred with ROSA26-Cre/ERT2 transgenic mice carrying the Cre-ER^{TM} gene driven by the endogenous mouse *Gt(ROSA)26Sor* promoter (B6.129-Gt(ROSA)26Sortm1(cre/ERT2)Tyj/J). To delete the floxed/floxed-Brg1 gene in developing embryos, ROSA-cre/ERT2 (B6; 129-Gt(ROSA)26Sortm1(cre/ERT2) Tyj/ Brg1$^{floxed/floxed}$ (Smarca4tm1Pcn) male mice mated to pregnant *Brg1*$^{floxed/floxed}$ dams were given 100mg/kg body weight pharmaceutical grade tamoxifen citrate (Sigma) IP at 12pm once daily for one day (E10.5 or two days (E12.5 and E13.5 stages). Embryos were harvested at different developmental stages, photographed and fixed in 10% neutral-buffered formalin for 24 h. Embryos were embedded in paraffin, sectioned at 5 um, mounted on glass slides and stained with hematoxylin and eosin for histological examination. For Brg1 genotyping, three primers were used to amplify wild type (241 bp PCR product; Primers: P1-P2), floxed/floxed (387 bp PCR product; Primers: P1-P2) and deleted (313 bp PCR product P1-P2-P3) Brg1 alleles; P1- GTCATACTTATGTCATAGCC, P2- GCCTTGTCTCAAACTGATAAG, P3- GATC AGCTCATGCCCTAAG. For Cre genotyping, the forward (5′- GCGGTCTGGCAGTAAAAACTATC-3′) and the reverse (5′- GTGAAACAGCATTGCTGTCACTT-3′) primer were used for PCR amplification [2]. All animal husbandry, handling, and experiments were performed in accordance with NIEHS/NIH guidelines covering the humane care and use of laboratory animals in research.

Tamoxifen dose determination

Tamoxifen dose determinations were described previously [1]. In brief, Tamoxifen for the 100 mg/kg dose was dissolved in ethanol to yield a 100 mg/mL stock, which was then diluted with corn oil to achieve a 10 mg/mL tamoxifen formulation (10% ethanol in final tamoxifen formulation).

Histology and immunohistochemistry

Timed mating was conducted using Cre/Cre; *Brg1*$^{floxed/floxed}$ and Cre/+; *Brg1*$^{floxed/floxed}$, *Brg1*$^{floxed/floxed}$ males with *Brg1*$^{floxed/floxed}$ females to obtain *Brg1*$^{floxed/floxed/Cre}$ and *Brg1*$^{floxed/floxed}$ embryos. The next day of mating females with vaginal plugs were considered to be at embryonic day 0.5 (E0.5) of gestation. Pregnant females were dosed with Tamoxifen (100 mg/kg body weight) and sacrificed at sequential time points of gestation (E6.5, E7.5, E8.5, E9.5, E10.5), and the embryos were dissected free of maternal tissue, examined, photographed, and genotyped by PCR. For histological preparation, embryos without decidua and in decidua were fixed in 4% paraformaldehyde–PBS or 10% neutral-buffered formalin for 18 h at 4°C, dehydrated, and embedded in paraffin. Paraffin-embedded tissues were sectioned at 5um, mounted on positively-charged glass slides.

Lyve1 staining

Immunohistochemical staining for Lyve1 was performed using the standard avidin-biotin-peroxidase technique. Formalin-fixed, paraffin-embedded tissue sections were deparaffinized in xylene and rehydrated through graded ethanol. Endogenous peroxidase blocking was done by immersing the sections in 3% H2O2 for 15 min after which heat-induced epitope retrieval was performed using a Citrate Buffer (Biocare Medical, CA) in the Decloaker® pressure chamber for 5 min at 120°C. Non-specific sites were blocked by incubating slides for 20 min with 10% normal donkey serum (Jackson ImmunoResearch, West Grove, PA), endogenous biotin and avidin binding sites were blocked using Avidin-Biotin Blocking Kit (Vector Laboratories, Burlingame, CA). The sections were then incubated with goat polyclonal Lyve1 antibody (sc-19319, SCBT, Santa Cruz, CA) at a 1:400 dilution for 30 min at room temperature. For negative control tissue section, a whole molecule goat purified IgG (Chrompure, Jackson ImmunoResearch, West Grove, PA), diluted to match the protein concentration of

the Lyve1 antibody was utilized. Secondary incubation was done using a biotinylated donkey anti-goat IgG antibody (Jackson ImmunoResearch, West Grove, PA) at a dilution of 1:500 for 30 min at room temperature. Labeling incubation was done with the Vectastain RTU Kit Label (Vector Laboratories, Burlingame, CA) for 30 min at room temperature. The antigen-antibody complex was visualized using 3-diaminobenzidine (DAB) chromagen (Dako, Carpenteria, CA) for 6 min, and counterstained with modified Harris Hematoxylin. The sections were then dehydrated through graded ethanol, cleared in xylene and coverslipped.

Endomucin and Prox1 Staining

Formalin fixed, paraffin embedded mouse tissues were deparaffinized and rehydrated. Endogenous peroxidase was blocked with 3% hydrogen peroxide. Antigen retrieval was performed with heat and pressure in a decloaking chamber, using a pH 6.0 citrate buffer retrieval solution (Biocare Medical, Concord, CA). The sections were incubated with 10% normal rabbit serum (Jackson Immunoresearch Laboratories, Inc., West Grove, PA) for 20 min, followed by the avidin-biotin blocking kit (Vector Laboratories, Burlingame, CA). For Endomucin staining, sections were incubated with Endomucin (Catalog#MAB2624, Millipore, Temecula, CA) monoclonal antibody (protein G purified IgG1) and purified rat IgG1 (negative control; BD Biosciences, San Jose, CA) for 60 minutes at 1:750 dilution. Prox1 staining was performed with Prox1 (Catalog # ab174244, Abcam) antibody for 1 h at 1:50 dilution. Sections were then incubated with a biotinylated rabbit anti-rat secondary antibody (Vector Laboratories, Burlingame, CA) for 30 min at 1:500 dilution. Label incubation was performed using Vectastain Elite ABC reagent, RTU (Vector Laboratories, Burlingame, CA) for 30 min also. Antigen-antibody complex was visualized using DAB (Dako, Carpinteria, CA) for 6 min. The sections were counterstained with hematoxylin, dehydrated, cleared and cover slip applied.

Quantitative RT-PCR analysis

RNA was isolated from early embryos using the Arcturus PicoPure RNA Isolation Kit and Invitrogen RNA Isolation Kit; cDNA was synthesized using the SuperScript First-Strand Synthesis System (Invitrogen) with Oligo dT primers. Real-Time quantitative reverse transcription PCR (qRT-PCR) measurements of individual cDNAs were performed with the SYBR Green Real-Time PCR detection system. Gene-specific primers for cell cycle regulators were designed for encoded gene transcript available at NCBI database using Primer Express. The rodent glyceraldehyde-3-phosphate dehydrogenase (*GAPDH*) was used as an internal control. Primer sequences are available upon request. All measurements were performed in triplicate. Values were normalized to *GAPDH* using the 2^{-DDCt} methods and expressed as ± SD. All mouse experiments were performed in accordance with NIEHS/NIH guidelines covering the humane care and use of animals in research.

Chromatin immunoprecipitation

Two litters of E8.5 mice were dissected in chilled PBS. ChIP was performed as previously described [4, 5] with minor modifications. Cells were cross-linked by adding 13.5 ul of 36.6% formaldehyde per 500 ul of sample for 15 min at room temperature. Fixation was stopped, by adding 57 ul of 1.25M Glycine to the sample. Cells were lysed in cold lysis buffer 1 (50 mM HEPES-KOH, pH7.5, 140 mM NaCl, 1 mM EDTA, 10% glycerol, 0.5% NP-40, 0.25% Triton X-100, 1x protease inhibitors) and gently rocked at 4°C for 10 min in 14 ml conical tubes. Cells were pelleted at 1350 × g at 4°C in a tabletop centrifuge, resuspended in cold lysis buffer 2 (10 mM Tris-HCl, pH 8.0, 200 mM NaCl, 1 mM EDTA, 0.5 mM EGTA, 1 × protease inhibitor), and gently rocked at 4°C for 10 min in 14ml conical tubes. Cells were pelleted at 1350 × g at 4°C in table top centrifuge and resuspended in 2 ml cold lysis buffer 3 (10 mM Tis-HCl, pH 8.0, 100 mM NaCl, 1 mM EDTA, 0.5 mM EGTA, 0.1% Na-Deoxycholate, 0.5% N-laurylsarcosine, 1 x protease inhibitors), and sonicated to 200–600 bp fragments using a Diagenode Bioruptor (3 × 10 minutes cycles, 30 s ON/ 30 s OFF at 4°C). For BRG1, and CHD4 ChIP, cells were resuspended and sonicated in sonication buffer (50mM Tris-HCl pH7.5, 140 mM NaCl, 1mM EDTA, 1mM EGTA, 1% Triton X-100, 0.1% Na-Deoxycholate, 0.1% SDS) for 15 cycles at 30 s each on ice (18W) with 30 s off on ice between cycles. Sonicated lysate were cleared by pelleting insoluble material at 20, 000 × g at 4°C followed by incubation with antibody bound Protein G magnetic beads (2.5 ug Ab/50 ul beads / IP) in 1 ml of 0.5% BAS/PBS overnight at 4°C. Magnetic beads were washed 3 times with block buffer (0.5% BSA/PBS), incubated for approximately 4 h at 4°C with antibody in block buffer, and then washed 3 times with block buffer prior to addition of cleared cell lysates. Chromatin was immunoprecipitated using BRG1 NB100-2594; NOVUS, and IgG. Immunoprecipitated material was washed five times with cold buffer (RIPA: 50mMHEPES-KOH, pKa 7.55, 500 mM LiCl, 1mM EDTA, 1.0% NP-40, 0.7% Na-Deoxycholate) and one time with TE plus NaCl 50 mM, followed by elution and reverse cross linking in 210 ul of 1% SDS in TE overnight at 65°C. 200 ul of reverse crosslinked material was treated with RNase A for 30 min to 2 h., proteinase K for 30 min to 2 h and extracted twice with phenol chloroform isoamyl alcohol, followed by ethanol precipitation with 3M sodium acetate and a glycogen co-precipitant, 80% ethanol wash and final resuspension in TE buffer or water. Nucleic acid yield was determined via Quant IT fluorescence assay (Invitrogen). Immunoprecipitated

chromatin was evaluated by qPCR (Stratagene Mx300P and Brilliant SYBR Green Quantitative PCR (QPCR) master mix). Average cycle threshold amplification values and percentage of sample input were calculated. The following PCR primers are used for QPCR analysis:

Lyve1 promoter sequence 1kb upstream and 1kb downstream from transcription start site (TSS) were downloaded from UCSC genome browser. Promoters of respective genes were analyzed for transcription factor binding using TRANSFAC – Gene Regulation software. Primers were designed from known transcription factor binding sites that potentially recruit BRG1.

Lyve1 primer location from TSS and putative transcription factor binding site: Forward (−) 436 bp; Reverse (-) 327 bp - Serum response factor (SRF) binding site (ATTTCCTTACA), Forward (+) 369 bp; Reverse (+) 480 bp – Coup-TFII binding site (TGGCAAAGGTG), ChIP primers used for Coup-TFII were described in a previous publication [3].

Promoter-Lyve1-F-M CAAAGGAAGGAGTGGT GCTT, Promoter-Lyve1-R-M GTACAAGCAGTGCCCA CCAC, Exon3-Lyve1-F-M CATCCCTCGGATTTTCT CAA, Exon3-Lyve1-R-M TCGGATGAGTTGTGGC AATA. Promoter-CoupTFII-F-M-CAAGCCTCGCCTTT AAGAAA. Promoter-CoupTFII-R-M- CAGCCCCAA ACAAGGTGTAA. Exon-CoupTFII-F-M-GCCTCAAAA AGTGCCTCAAA. Exon-CoupTFII-R-M- AGGCATCC TGCCTCTCTGTA

Cell lines and cell culture

Human Umbilical Vein Endothelial Cells (HUVEC) were obtained from the Thermal Fisher Scientific and cultured according to the instructions of the manufacturers. 2×10^5 HUVEC cells were seeded per well in a 6 well plate 24 hrs prior to transfection with siRNA. Next day cells were transfected with 100 pmol of either NT control or BRG1 siRNA using lipofectamine protocol according to manufacturer's instructions. Controls with lipofectamine and no lipofectamine reagent were also included. During transfection cells were maintained in complete 200PRF media supplemented with low serum growth supplement (Thermal Fisher Scientific). For endothelial cell tube formation, 24 hr post transfection cells were trypsinized and counted. 40,000 cells from each siRNA treatment were seeded on Geltrex coated well on a 24 well plate and maintained, in complete supplemented 200PRF media as described for endothelial cell tube formation assay (Thermal Fisher Scientific). Cells were incubated on the Geltrex matrix for 6 hrs and images acquired. Cells were incubated on the Geltrex matrix for 16hr and the stained with 2 ug/mL Calcein AM (Cat No C3099, Thermal Fisher) for 30 minutes followed by imaging.

SW-13 human adrenal carcinoma cells were maintained as described previously [4]. Transfections were carried out in antibiotic-free Dulbecco's modified Eagle high-glucose medium by using Lipofectamine® 2000 Transfection Reagent according to the manufacturer's instructions (Life Technologies, Thermo Fisher Scientific, Waltham, MA). Briefly, 2×10^5 cells were transiently transfected with 1 ug of either control empty vector (pcDNA3.1) or BRG1 expression plasmid (pcDNA3.1/BRG1–3 × Flag), as previously described [4]. Twenty-four hr post transfection, cells were harvested for protein and total RNA extraction as described previously [1].

REFERENCES

1. Singh AP, Foley JF, Rubino M, Boyle MC, Tandon A, Shah R, Archer TK. Brg1 Enables Rapid Growth of the Early Embryo by Suppressing Genes That Regulate Apoptosis and Cell Growth Arrest. Mol Cell Biol. 2016; 36:1990–2010. doi: 10.1128/MCB.01101-15.
2. Sumi-Ichinose C, Ichinose H, Metzger D, Chambon P. SNF2beta-BRG1 is essential for the viability of F9 murine embryonal carcinoma cells. Mol Cell Biol. 1997; 17:5976–86.
3. Davis RB, Curtis CD, Griffin CT. BRG1 promotes COUP-TFII expression and venous specification during embryonic vascular development. Development. 2013; 140:1272–81. doi: 10.1242/dev.087379.
4. Trotter KW, King HA, Archer TK. Glucocorticoid Receptor Transcriptional Activation via the BRG1-Dependent Recruitment of TOP2beta and Ku70/86. Mol Cell Biol. 2015; 35:2799–817. doi: 10.1128/MCB.00230-15.

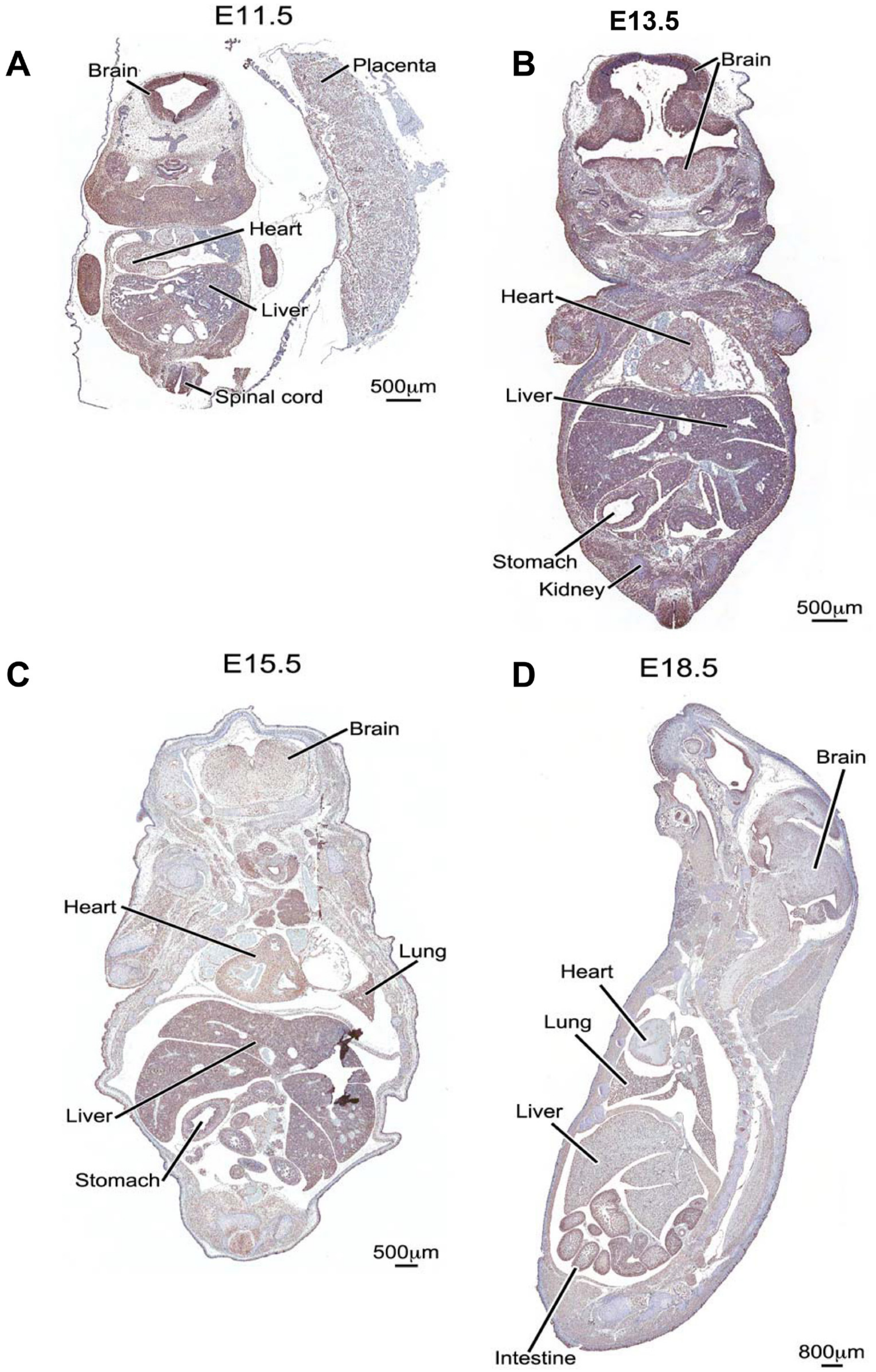

Supplementary Figure 1: Spatiotemporal localization of BRG1 in wild-type embryos during mid-gestation period of development. (**A–D**). Histological sections of mouse embryos from different developmental stages E11.5, E13.5, E15.5 and E18.5 show strong BRG1 immunostaining in all the organs and tissue types including brain, heart, liver and intestine. The stain was developed using 3-diaminobenzidine (DAB) substrate (Dakocytomation, CA) and then counterstained with modified Harris Hematoxylin. DAB binds to the areas where antibody-antigen interaction has occurred (brown color). The regions of the cell that do not have a DAB reaction are covered by the counterstain (Harris Hematoxylin; blue color).

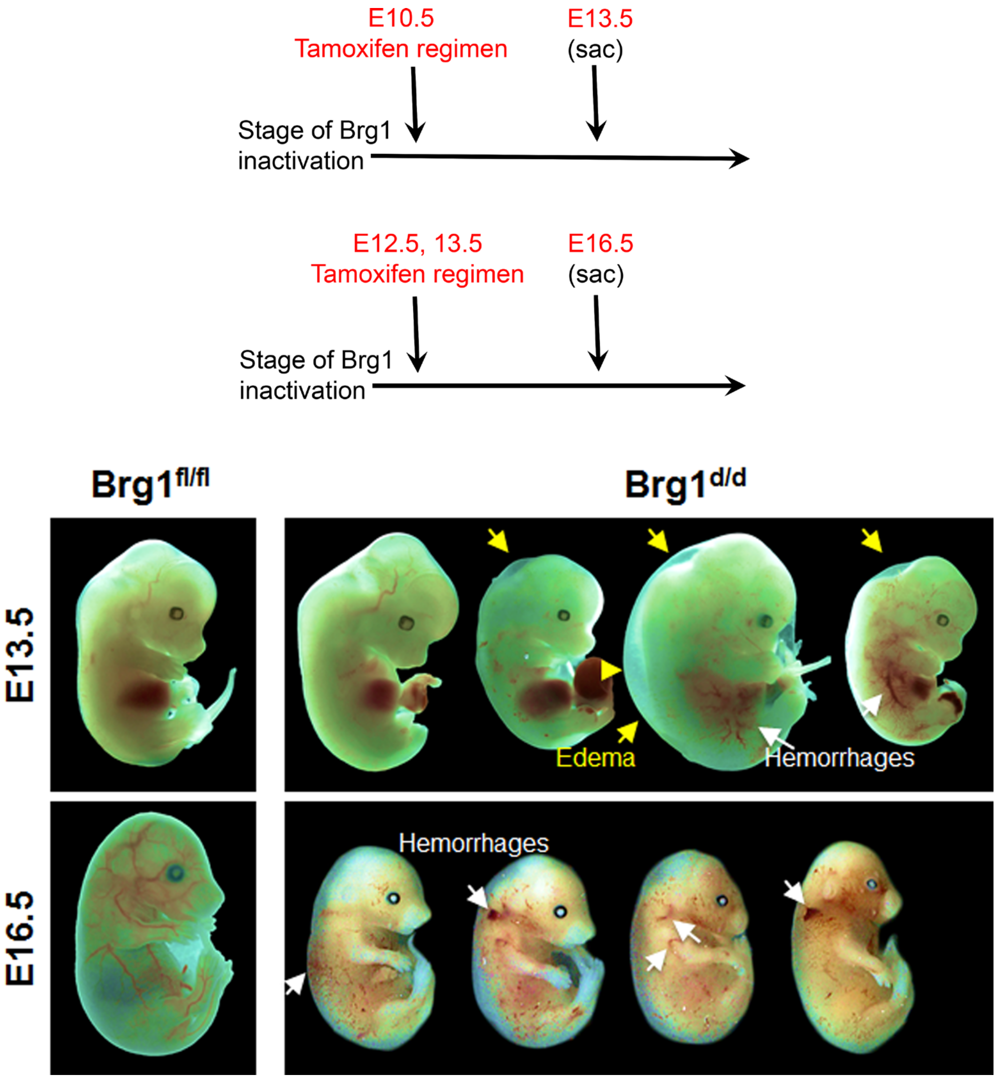

Supplementary Figure 2: *Brg1*$^{d/d}$ embryos exhibit hemorrhages. Pictures show gross morphology of the *Brg1*$^{fl/fl}$ and *Brg1*$^{d/d}$ embryos at the indicated developmental stages. Yellow arrows–edema; white arrows– hemorrhage. Schematic shows developmental stages of tamoxifen dosing and embryo collection (lower panel).

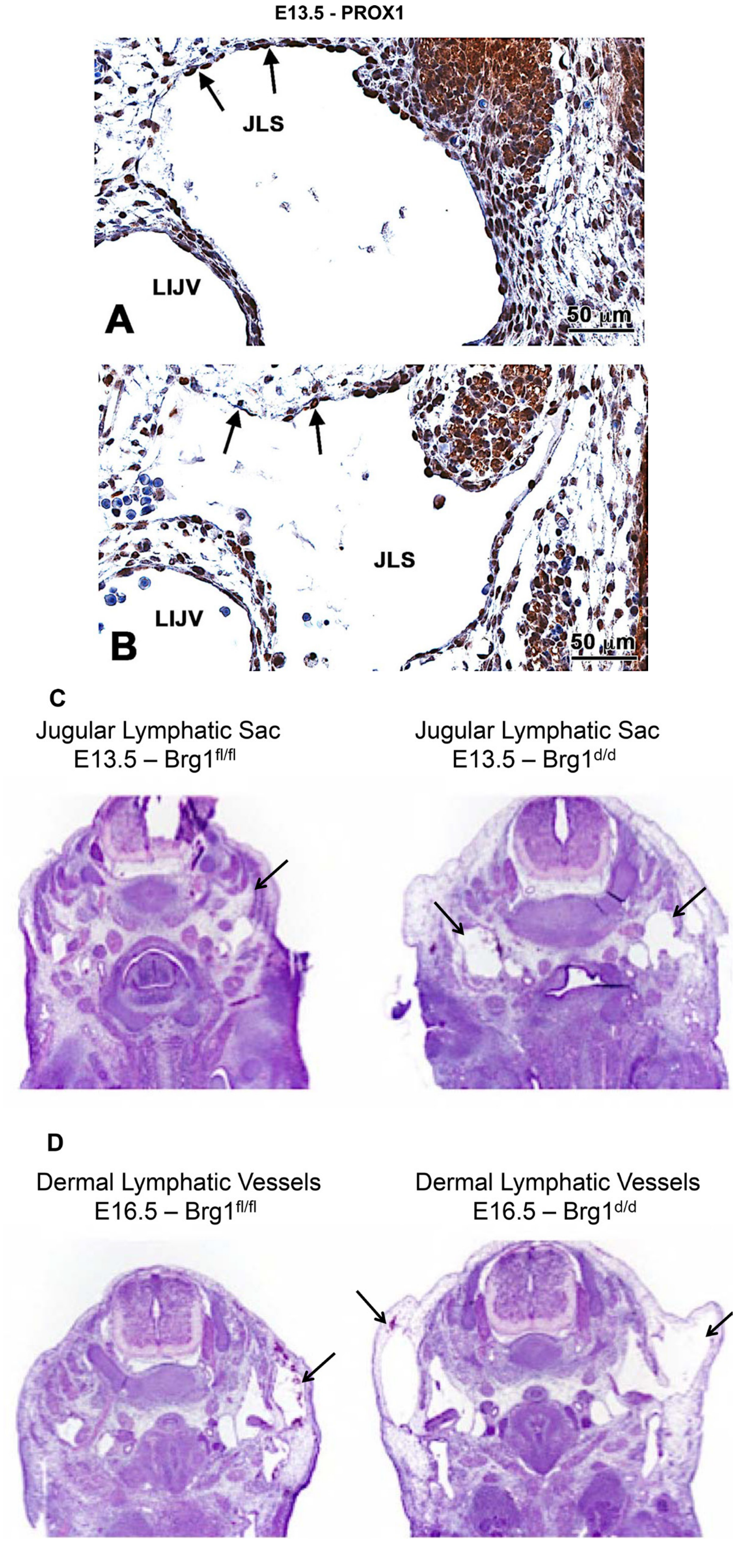

Supplementary Figure 3: Immunohistochemistry detects distribution of Prox1 in *Brg1$^{fl/fl}$* and *Brg1$^{d/d}$* embryos. Immunohistochemical staining for PROX1 identifies the lymph sacs (ls) in E13.5 *Brg1$^{fl/fl}$* and *Brg1$^{d/d}$* embryos. PROX1 staining is comparable in *Brg1$^{fl/fl}$* and *Brg1$^{d/d}$* embryos. CS, carotid sinus, JLS, jugular lymphatic sac, LIJV, left internal jugular vein.

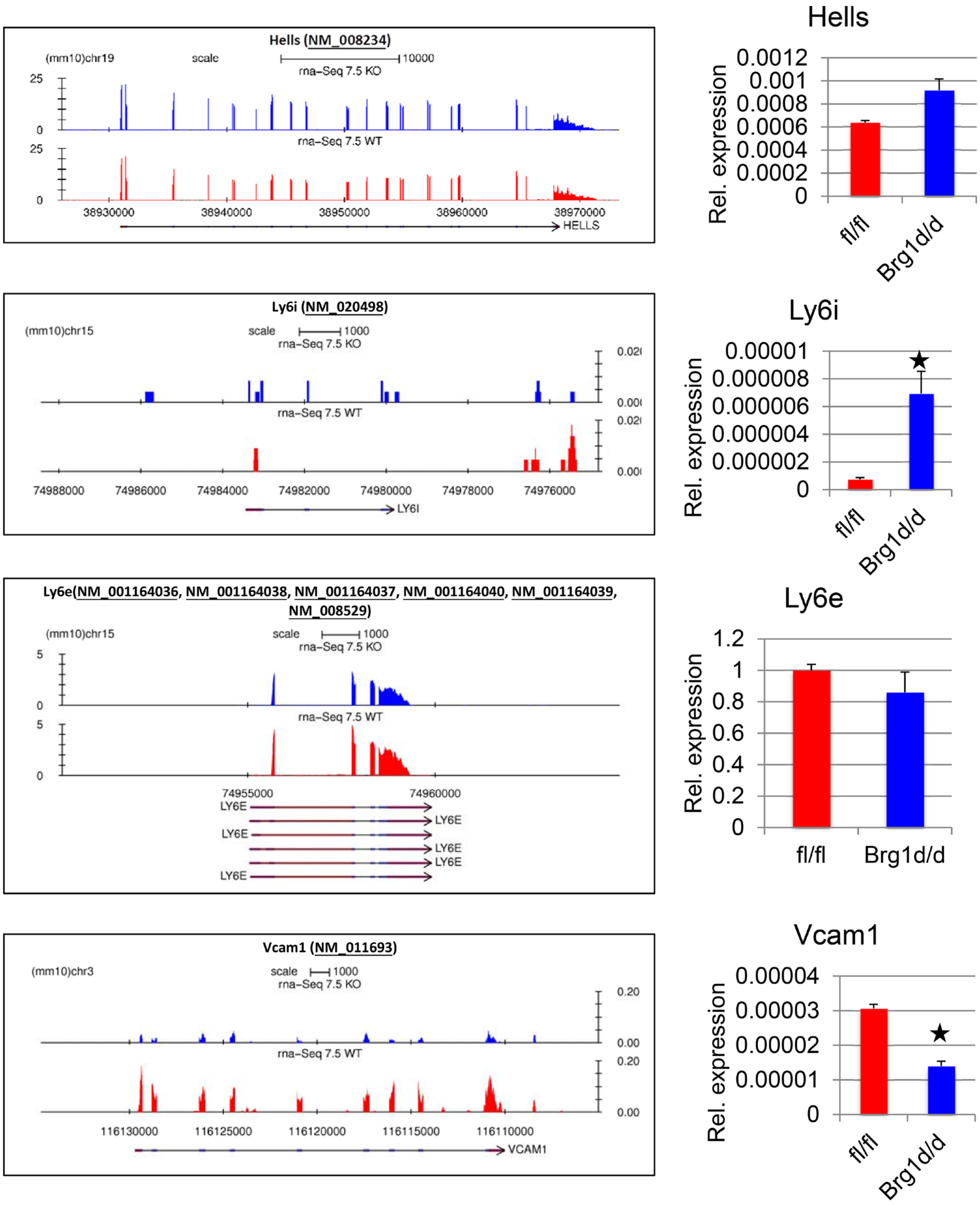

Supplementary Figure 4: RNA sequencing and QPCR expression of selected genes in *Brg1*$^{d/d}$ vs. *Brg1*$^{fl/fl}$ embryos. Left panel, RNA seq. analysis shows gene track and RNA expression of the selected genes in *Brg1*$^{fl/fl}$ and *Brg1*$^{d/d}$ E8.5 embryos. Right panel, quantitative RT–PCR shows mRNA level of the Hells, Ly6i, Ly6e, and Vcam1 in *Brg1*$^{fl/fl}$ & *Brg1*$^{d/d}$ E8.5 embryos.

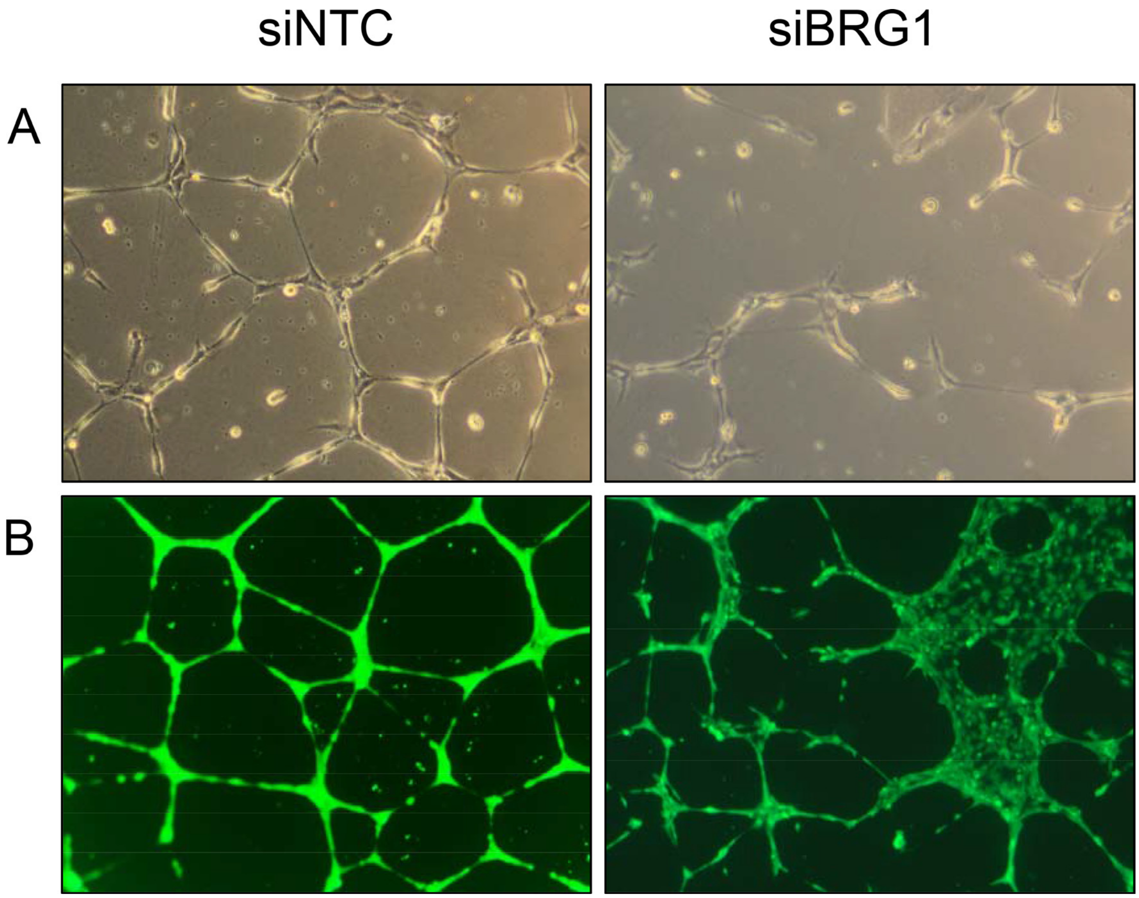

Supplementary Figure 5: Human vascular endothelial cells (HUVEC) were transfected with siRNA of non-targeting control and BRG1. HUVEC cells were trypsinized after 24 hrs of transfection and reseeded on GELTREX for tube/vessel formation. Bright field (**A**) and fluorescent (**B**) images of HUVEC cells formed tube/vessels stained with Calcein for 30 minutes. Images were taken at ~16–18 hrs after seeding on GELTREX.
